# Stable Millivolt Range Resistive Switching in Percolating Molybdenum Nanoparticle Networks

**DOI:** 10.1021/acsami.4c12051

**Published:** 2024-11-13

**Authors:** Adrianus Julien Theodoor van der Ree, Majid Ahmadi, Gert H. Ten Brink, Bart J. Kooi, George Palasantzas

**Affiliations:** † Zernike Institute for Advanced Materials, University of Groningen, 9747 AG Groningen, The Netherlands; ‡ CogniGron Center, University of Groningen, 9747 AG Groningen, The Netherlands

**Keywords:** Brain-like networks, nanoparticle networks, percolation, long-range temporal correlations, neuromorphic computing, scanning transmission electron microscopy, low power

## Abstract

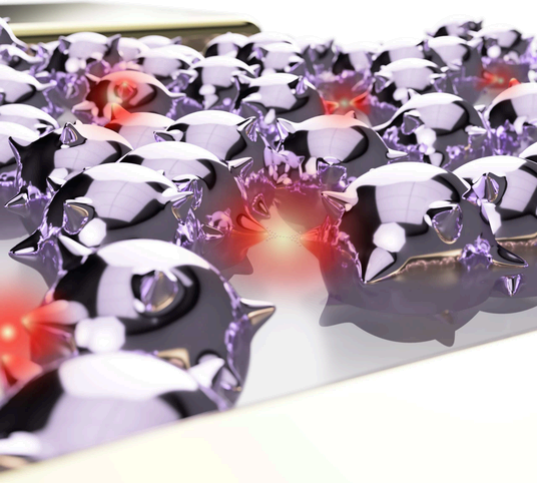

To overcome the limitations of the conventional Von Neumann architecture, inspiration from the mammalian brain has led to the development of nanoscale neuromorphic networks. In the present research, molybdenum nanoparticles (NPs), which were produced by means of gas phase condensation based on magnetron sputtering, are shown to be the constituents of electrically percolating networks that exhibit stable, complex, neuron-like spiking behavior at low potentials in the millivolt range, satisfying well the requirement of low energy consumption. Characterization of the NPs using both scanning electron microscopy and scanning transmission electron microscopy revealed not only pristine shape, size, and density control of Mo NPs but also a preliminary proof of the working mechanism behind the spiking behavior due to filament formations. Furthermore, electrostatics COMSOL Multiphysics simulations of the morphology of the NPs provided evidence that the stable switching is due to the as-deposited stellate Mo NPs creating high electric field strengths, while keeping them separated, not seen before in other percolating networks based on spherical NPs. Hence, our results show the working mechanism behind switching in percolating Mo NP networks and show that they are very promising for realistic neuromorphic systems.

## 1 Introduction

Despite their astounding success in modern world technology, conventional computing architectures face fundamental limitations. The Von Neumann bottleneck, the end of Moore’s law, and the increasing power consumption scream for new, competing, high-density, and energy-efficient computing architectures. Neuromorphic or ’brain-like’ computing seeks to overcome the aforementioned limitations by physically emulating the neuronal and synaptic building blocks of the energy-efficient^1,2^ mammalian brain at the device level. Neuron- and synaptic-like behavior, which has been emulated with traditional transistor-based circuits, is still yielding similar limitations.^3,4^ As of yet, neuromorphic systems have been created using electrochemical organic devices,^5^ memristors,^6−8^ memtransistors,^9^ phase-change memory devices^10^ and complex networks of nanowires,^11,12^ and nanoparticles (NPs).^13−22^

The latter have recently emerged as an alternative for neuromorphic computing systems. Percolating NP networks in particular have been utilized as they exhibit electrical spiking signals comparable to networks of biological neurons.^20^ This behavior has been attributed to the vast amount of tunneling gaps and filamentary bridging of such gaps between well- and poorly connected NP clusters, due to electric field-induced surface diffusion.^23^ This, in essence, is a random network of break-junctions, often undesirable in electronics, that are now being functionalized. The atomic hopping is visualized in electrical conductance measurements, where clear, multilevel switching is observed.^14,24,25^ Albeit differently sized filaments, similar filamentary switching dynamics have previously been imaged using conductive atomic force microscopy and holography transmission electron microscopy (TEM).^26,27^ Recently, *in situ* TEM electrical measurements have been performed on Au NP networks, where Joule heating was found to act as the driving force behind resistive switching.^28^ As of yet, no filaments in percolating NP networks have been observed directly yet. Finally, among other application-oriented experimental works,^17,22,29^ percolating NP networks have been shown to be able to perform Boolean operations and image classification, providing a basis for functional NP network devices.^30^

So far, gold, tin, bismuth, and silver have been the choice of the materials used in the NP networks, showing similar neuron-like spiking behavior.^13,15,21,23,31^ These NP networks are, however, biased at levels ranging from 1 to 40 V to induce resistive switching.^14,16,20,21^ Such large bias voltages are suspected to be due to the large electrode separations and the inherent NP morphology. Nevertheless, the bias levels and their accompanying power necessary for switching are fairly high and seem unrealistic if advances in energy-efficient neuromorphic computing are to be made with operating voltages well below 1 V.

Here, we present percolating Mo NP networks capable of room temperature, resistive switching at applied bias voltages as low as 1 mV. Performing *in situ* measurements to reach percolation and long-duration, stable measurements at sub-50 mV biases have revealed neuron-like switching behavior. COMSOL Multiphysics simulations, paired with a comparison with copper based percolating NP networks, show evidence of concentrated electric fields at the spike-like features of the as-deposited stellate Mo NPs facilitating electromigration well and enabling low bias, stable switching. Furthermore, utilizing (scanning) transmission electron microscopy ((S)TEM), the threshold of percolation has been confirmed, energy dispersive X-ray spectroscopy is used to reveal reasons for nonquantized steps, and initial confirmation of filament formation in between the spike-like features of the NPs has been found. Moreover, the observed filament possesses a crystallographic structure that differs from that of the produced Mo NPs.

## 2 Results and Discussion

**Figure 1:**
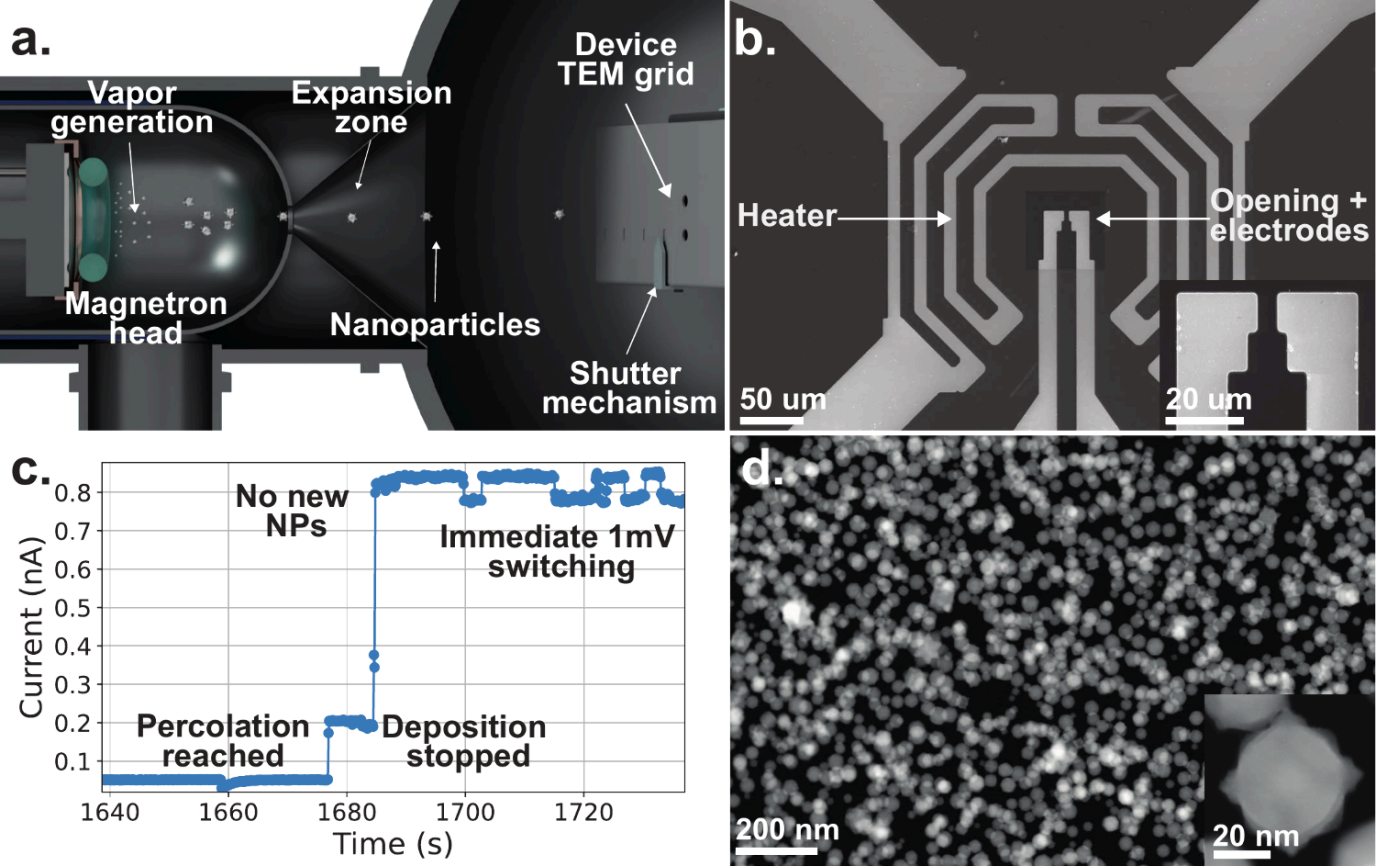
**a.** Schematic of NP production via gas-phase synthesis (not to scale). Utilizing a magnetron sputterer and an aggregation chamber, NPs can be produced and deposited onto an electrically monitored (1 mV bias), (S)TEM-compatible chip, as seen in **b.** In **c.**, the electrical conductivity of the chip is monitored so that one can identify the point of percolation (the first substantial rise in conductivity) and halt the deposition. Note the immediate switching under a 1 mV bias voltage after halting the deposition. A pristine size and deposition rate control yields a percolating network of homogeneously sized NPs seen in **d.**, the HAADF (S)TEM image of a percolating NP film, with a covered area of 67 ± 1%. The inset shows a typical, as-deposited, stellate Mo NP.

**Figure 2:**
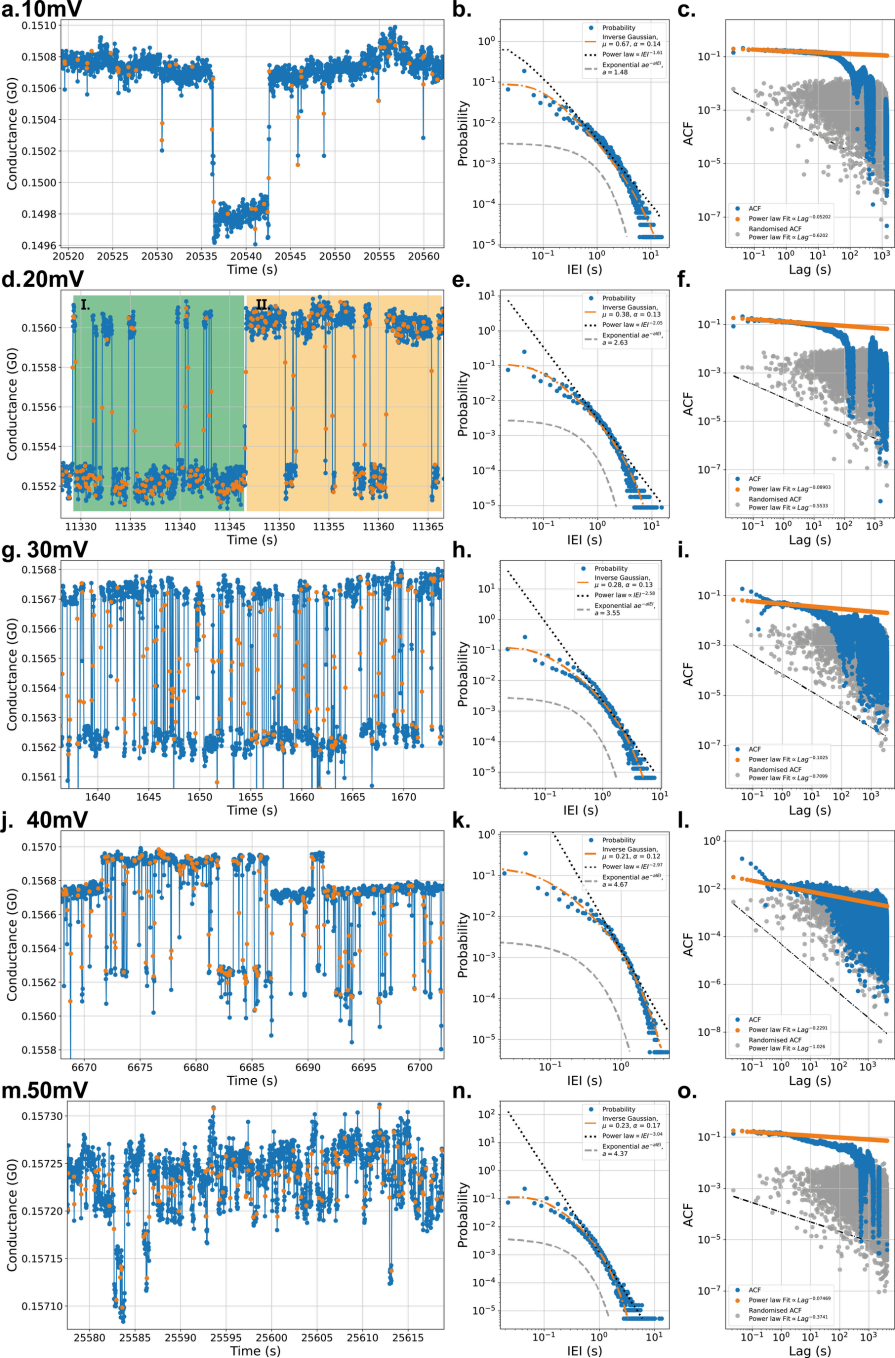
Five 12-h continuous measurements of the same NP network at 10 (**a.**), 20 (**d.**), 30 (**g.**), 40 (**j.**), and 50 (**m.**) mV biases have been performed with respective zoomed-in sections of approximately 35 s shown in the first column. The switches have been marked in orange. Note the increase in switching frequency upon increasing the bias application. In **d.**, levels are sometimes predominantly occupied with switching occurring rapidly toward the other level highlighted in sections **I.** and **II.** This occurs in all bias applications. In the second column (**b.**, **e.**, **h.**, **k.**, and **n.**), the respective (heavily tailed) PDF distributions of the IEIs are shown, with binning equal to the measurement interval. A comparison is made between three different fitting models: an inverse Gaussian fit in an orange dash-dot line; a power law fit in a black dotted line; and an exponential fit in a gray dashed line. Finally, in the last column (**c.**, **f.**, **i.**, **l.**, and **o.**), the respective autocorrelation functions (ACFs) are shown. In blue, the ACFs with its orange power law fit are shown. Upon shuffling the IEI sequence, correlations are lost, as seen in the gray ACF with its accompanying, black-dotted power law fit. Note: all fits are within a 95% confidence interval. The identification thresholds are 8, 5, 7.5, 5, and 2 × 10^–5^ for the data sets of 10–50 mV, respectively.

When percolation is achieved and deposition is halted (Figure 1c), the application of a constant bias voltage can induce switching activity. Figure 2 shows results, where 10, 20, 30, 40, and 50 mV biases were applied over 12 h. More snapshots of the full data set can be seen in Figure S2. Switching activity can be observed clearly in the zoomed-in sections in Figure 2a, d, g, j, and m. The switches, identified with the method mentioned in the methods section, have also been indicated with orange dots. The switches do not occur periodically and hence are intrinsically stochastic.

The five different bias values result in systematic differences in the switching dynamics. Switching of this NP network does not take place at voltages below 5 mV. However, upon an increase beyond 5 mV, switching starts to occur. Upon increasing the voltage bias even more, the switching frequency will typically increase, due to (de)construction of the filamentary bridges happening faster and at a greater number of poorly connected NP clusters. Switching behavior where clear differences occur between long-lived and short-lived resistance levels can also be observed. For example, in Figure 2d, the two highlighted regions indicate the conductance levels dominantly occupied by either the lower or upper level, with short-lasting switches to the upper or lower level, respectively. Both of these temporally different switch events occur in all bias applications. Such longer lived states are indicative of a filamentary bridge having formed in the network where a low amount of current is passing, Joule heating this filament slowly, or where the electric fields are less concentrated, slowing electromigration for the formation of a filament. Upon increasing the bias to 50 mV however, the network showed such rapid switching that we believe there are switches not measured with our current measurement rates. Nonetheless, these percolating NP networks are believed to be temporally self-similar.^19^

The resistive switching can be attributed to inter-NP electric field changes, changing electron densities, and currents in the networks. This results in electromigration of atoms, but also Joule heating, rearranging atoms into a state where a single atom is jumping between the respective NPs.^24,25,32,33^ The local electric fields affect the atom and network arrangements, albeit by electromigration or Joule heating. The changes in occupied level and switch duration can be influenced by external factors (e.g., electric fields or temperature) and could have the potential to be used as stochastic neuristors, comparable to the work of Acharya et al.^33^

Train spike analysis was performed to further analyze stochastic and avalanche behavior based on the switching times. In Figure 2b, e, h, k, and n, the respective PDFs of the IEIs are shown, where underlying power law behaviors are often associated with correlated avalanches.^19,33^ This burst, avalanche-type behavior is also confirmed by the heavy-tailed PDF. The two-level fluctuations in Figure 2 also appear to be rather similar to random telegraph noise. To confirm that the accompanying exponential statistics are nonexistent, an exponential fit is also plotted to show that this system possesses different dynamics. In these cases, however, neither a power law nor exponential dynamics represent sufficiently well the switching dynamics. The latter is better represented by an inverse Gaussian distribution due to its stochastic nature. Power law distributions are believed to be slightly lacking due to the lack of faster measurements, revealing the faster switch events. To further analyze avalanching effects, ACFs can provide initial evidence for strong correlations. In the last column in Figure 2, the ACFs are represented. Here, the blue data sets represent the ACFs of the IEIs and are fitted with a power law in orange. To observe differences with a completely uncorrelated data set, the IEI sequence is randomly shuffled and is represented in gray, with a black dotted power law fit. In all cases, the slope of the ACF is significantly smaller, indicating avalanching with long-range temporal correlations. Directly measuring ACFs tends to be difficult in the neuroscience field ,and hence, the Hurst exponent can act as a measure for correlated activity in percolating NP networks.^19^ Estimating the Hurst exponent (*H*) is done via the following formula: β = 2 – 2*H*, where β is the exponent of the power law fit of the ACFs. For the measurements performed here, the Hurst exponents are estimated to be 0.9, significantly above a Hurst exponent of 0.5, typical for uncorrelated processes, and thus indicating highly correlated activity. We note, however, that further experiments, such as multielectrode arrays, could give harder evidence for such correlations throughout the network, more typically found in neuroscience community.^34^ Moreover, because of the low biases and currents, the measuring rate cannot be increased without losing accuracy or precision, which might skew our results toward the inverse Gaussian distribution.

Quantized conductance switching is not observed upon examination of the step sizes in Figure S6. Here, the distribution of the sizes of the switches shows the changes in conductance range over approximately 2 orders of magnitude. Different switch sizes indicate different filamentary bridges being formed at different locations in the network. The distributions, however, do not closely follow the power law fit due to the steps not being distributed over a range. Rather, since the device geometry is relatively small, there are only a few locations in the percolating NP network where switching occurs. This means that the switch sizes are a bit more defined rather than a power law distribution. As stated above, the 50 mV bias application likely pushes the network into a rapid switching state which is difficult to measure and analyze. This is also revealed in the probability distribution in Figure S6, confirming our reasoning above. Analyzing the total measurement, one can see in Figure S6 that steps of approximately 1000ths of G0 are observed. The reason why the steps are not quantized can be attributed to a very thin oxide shell around the Mo NPs, contrary to previous work.^35^ The filaments formed could then possess an oxide crystal structure, allowing for an electron transmission probability of less than 1. Even though filaments are still formed and broken, quantized conductance will not be observed. Moreover, the measurements do not just span single filament formations or destructions in the network. The complete network is being measured, which, similarly to resistors in series and parallel, prohibits us from seeing quantized conductance.

**Figure 3:**
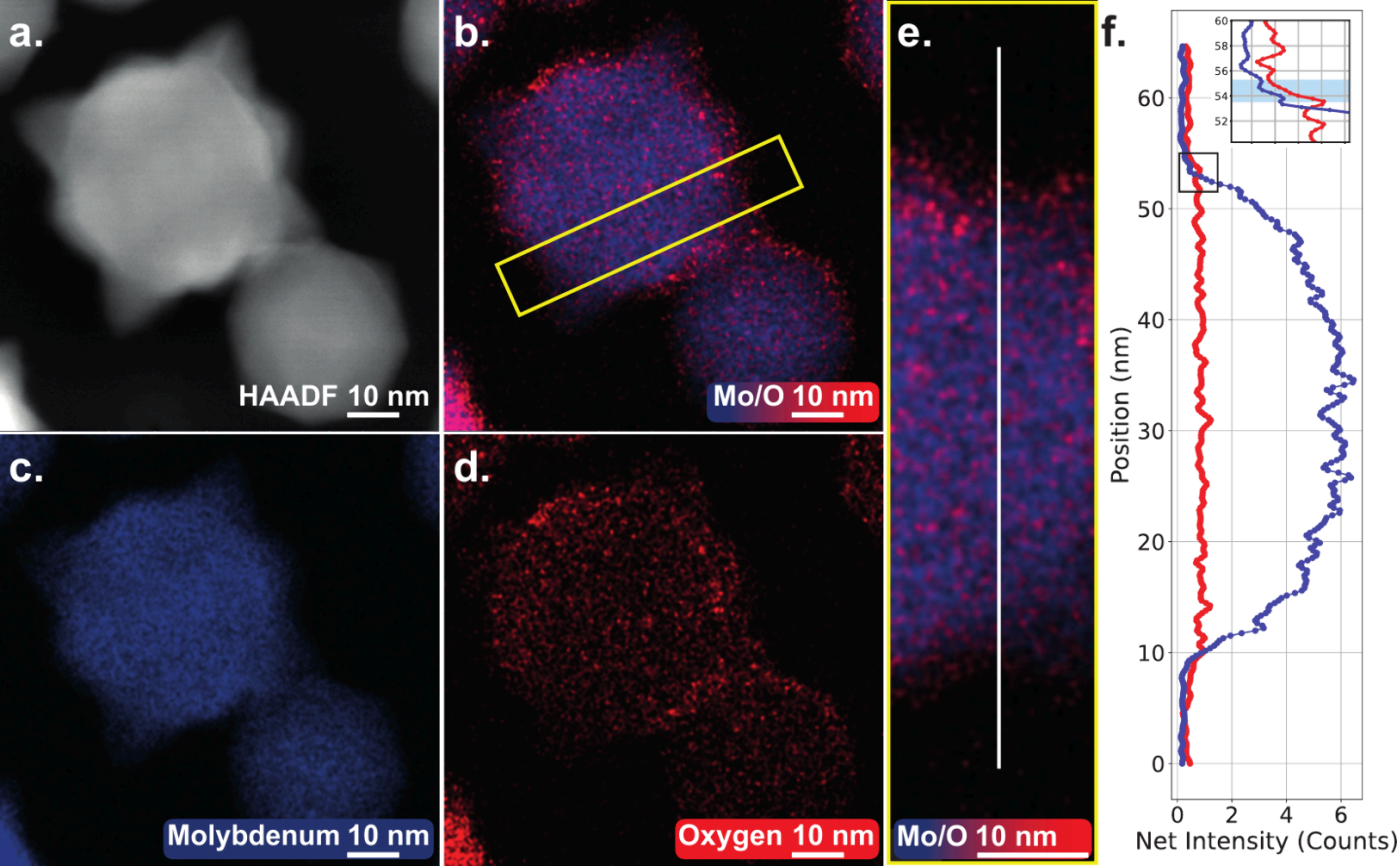
Energy dispersive X-ray spectroscopy (EDS) of a larger (≈40 nm in diameter) Mo NP with a thin oxide shell (1–2 nm). **a.** Shows a HAADF-STEM image overview where in **b.** the STEM-EDS combined color maps reveal the distribution of molybdenum and oxygen. **b.**, **c.**, and **d.** are color maps based on spectrum imaging; i.e., for each pixel an EDS spectrum is measured. Clear distinctions between respective elemental concentrations can be seen. Paired with **e.** and **f.**, a sectional line profile, one can see a clear rise of oxygen at the edges of the NP, indicating shell oxidation of approximately 1–2 nm as highlighted in the inset in blue.

Performing (S)TEM electron dispersive X-ray spectroscopy (EDS) on a stellate Mo NP in Figure 3 reveals oxidation is likely the cause for the nonquantized conductance steps, as a thin (1–2 nm) oxide shell is observed. Here, an elemental mapping of a Mo NP is made directly after deposition. The elemental mapping with a sectional line profile shows pristine Mo NPs with a thin oxide shell, contrary to previous work.^35^ The oxidation does not influence the working mechanisms of the percolating network in a negative sense, as switching continued for long durations of time, without showing any signs of failure. An oxide shell is even beneficial as it hinders the coalescence of NPs, which would prohibit the presence of poorly connected clusters of NPs and, thus, would omit filamentary formations.

To investigate the influence of the morphology on electric field distribution, a COMSOL Multiphysics electrostatics simulation was set up for our stellate NPs and compared with purely spherical NPs; see the methods section for more details. In Figure S8, electrostatic simulations of differently oriented NPs are shown. Using two cross-sectional planes, streamlines, and NP surfaces, relatively high electric field strengths between the biased NPs are highlighted in red concentrated areas. Compared to spherical NPs, one can observe large differences in both magnitude and distribution of the electric fields. The features of the NPs result in high electric field strengths at the tips in all orientations (see supporting movie Figure S9), and they can facilitate electromigration well at lower voltage biases. Moreover, electromigration will therefore be dominant at the tips compared to the bulk of the NP, more so than spherical NPs. One could argue that spherical NPs are, therefore, also easier to coalesce and create more permanent connections due to the amount of material which might electromigrate and hereafter need to be Joule heated to break apart again. By comparison of these two morphologies, stellate NPs can have more consistent switching dynamics.

**Figure 4:**
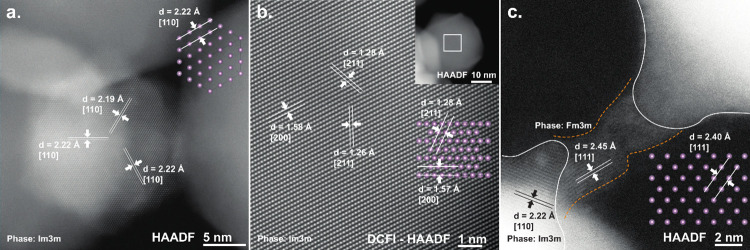
**a.** HAADF (S)TEM image of a Mo NP where the [110] planes can be identified. **b.** Drift corrected frame integrated (DCFI)-HAADF (S)TEM image of a Mo NP (inset) where the [211] and [200] planes can be identified. **c.** (S)TEM image of two Mo-NPs where a filament has been formed between the two tips of the NPs due to biasing the device. The respective insets represent the Im3m body-centered cubic (bcc) crystal structures in their respective orientations. Although the filament crystal structure fails to match the bcc crystal structure of the NPs, it does match the Fm3m face-centered cubic, Mo crystal structure.

One different aspect of the percolating Mo NP networks compared to previous works is the switching dynamics. As can been seen in Figure 2, the switching is predominantly 2-level switching. This switching is very stable and, even over 12 h, still extremely close to its original starting point. We believe, when compared to the works of Fostner and Brown,^15^ Sandouk et al.,^16^ and Mirigliano et al.,^21^ this behavior can be attributed to the significant difference in our NP morphology compared to theirs, represented in Figures 1d, 3, 4, and S7. Characteristic for our as-deposited NPs is that they are stellate,^35^ whereas in these earlier works, the as-deposited NPs are spherical. This morphology aids in keeping NPs apart and aids in reducing coalescence. This results in stable, consistent, and different switching dynamics. To gain insight into material differences, spherical, 12 nm diameter Cu NPs were the constituents of a different percolating NP network. Switching in percolating Cu NP networks required similar bias applications to induce switching. This does not bode well for the low-bias switching claim of Mo NP networks. However, there were two key differences. The Cu networks would regularly have permanent, large switch events, indicative of melting of the network, and the average conductance would not stop to increase (even after significant efforts to omit this behavior), indicating substantial coalescence of the network (see Figure S10). This is because both shape and material, due to coppers relatively low bonding energy (comparable to previous works), make coalescence and melting of the network significantly easier. Moreover, the comparatively low bonding energy can also lower the bias threshold for switching to occur, as electromigration now requires less energy. However, since the threshold for switching activity was similar and the sizes of the Cu NPs are smaller, compared to the Mo-based NP networks, device geometry or materials from previous works should be explored. Nonetheless, we believe that the stellate shape of the Mo NPs, as well as the material difference itself, aids in the stable, low bias, resistive switching due to the differences with copper NP networks.

Until recently,^28^ no direct confirmation of the switching mechanisms in percolating NP networks has been reported. The switching mechanisms in previous works have so far been attributed to filamentary formation between poorly connected NP networks or even to alignment of mismatched crystalline NP orientations^21^ and depercolation of the network.^28^ Preliminary confirmation of filamentary switching between poorly connected NP networks is observed in Figure 4c. This is because the fragile, electrostatic discharge sensitive device was inoperable after insertion into the microscope. In Figure 4a and b, two different Mo NPs have been imaged to extract the crystal structures. Both reveal the well-known body-centered cubic (bcc) crystal structures. Although electromigration caused the formation of the filament in Figure 4c, the filament did not grow according to the crystal lattice of the parent NP(s). On the contrary, its structure is rather face-centered cubic (fcc). Interestingly, earlier work has shown that when the dimensions reduce to approximately 2.5 nm, their crystal structure switches from bcc to fcc, which can be attributed to the more dominant role of surface energy in determining the equilibrium structure of the NPs when they become smaller, which favors fcc over bcc.^36^ However, earlier work has not yet revealed that stable fcc Mo can be observed in the vicinity of bcc Mo. Moreover, in Figure 4c, one can also observe that the filament has been formed in between two tips of the neighboring stellate Mo NPs, confirming that ultralow bias voltage switching, with accompanying low power, in this percolating Mo NP network is due to the morphological differences of the as-deposited stellate NPs compared to previous works based on spherical NPs.

## 3 Conclusion

In conclusion, percolating molybdenum nanoparticle networks, which exhibit filamentary switching, have been produced. Switching manifested in the form of filamentary bridges, which can also be heavily influenced by changing the input bias voltages. This process is enabled not only by an oxide shell surrounding the Mo NP but also by the stellate morphology of the NPs, responsible for stable and consistent switching. Further electrical analysis reveals avalanching behavior, which is comparable to brain-like activity. The voltage bias to necessitate switching activity, however, lies in the millivolt range and is much lower compared to previous works. We have provided evidence to explain this behavior using COMSOL Multiphysics simulations indicating that this is due to morphology differences between the as-deposited stellate Mo NPs and the spherical-like NPs from earlier works. By comparing Mo NP networks with a copper based percolating NP network, where a similar bias application threshold for switching was found, we can conclude that morphology and material choice definitely are very important: Cu NP networks show coalescence unlike the Mo NP based networks, which is omitted in the Mo network due to the NPs’ stellate shape. Furthermore, by utilizing electron microscopy, we show that a network at precisely the percolation threshold has been achieved and that the working mechanism behind the switching is indeed filamentary bridging in between spike-like features of the Mo NPs, enabling low bias voltage switching percolating NP networks. The filament shows an fcc crystal structure, deviating from the bcc structure within the Mo NPs, which can be attributed to the dominance of minimization of surface energy. The fragile devices are, however, quite prone to electrostatic discharge when using *in situ* atomic resolution electron microscopy, and a future solution must be implemented to fully confirm filamentary formation and breakage between poorly connected NP networks. The stellate NPs facilitate electromigration and filament formation well compared to spherical NPs and thus provide a gateway to more energy-efficient but, more importantly, reliable and stable neuromorphic devices based on percolating NP networks.

## 4 Methods

### 4.1 Production of Nanoparticles

Mo NPs are deposited onto a (S)TEM-compatible chip (5 μm spacing, 10 μm wide electrodes) by a home-modified Mantis Ltd. Nanogen 50 NP deposition system which utilizes high-pressure magnetron sputtering of a Mo target (50.8 mm diameter, 3 mm thickness, and purity of 99.99%) under Ar gas flow (purity 99.9999%), a low concentration CH_4_ reducing gas^37^ (2 ppm), and a 0.70 A discharge current, to produce monodisperse (20 ± 2 nm) Mo NPs (see Figure 1). The base pressure of the system was 1 × 10^–8^ mbar, where the reducing gas CH_4_ is introduced to increase the NP yield and reduce deposition time yet not influence the purity or quality of the NPs. The pressure in the main chamber (where the (S)TEM-compatible chip resides) and the aggregation chamber, under operation, is 5 × 10^–4^ mbar and 5 × 10^–1^ mbar, respectively. To produce percolating NP networks, *in situ* electrical measurements were performed during NP deposition.

### 4.2 Electrical Characterization

*In situ* electrical characterization is performed initially to monitor the sample until percolation is reached, upon which the deposition of NPs is terminated. Then long-term electrical characterization of the percolating NP network is performed using a Keithley 2602A source-measuring unit (SMU) paired with a homemade Python software to control the SMU. A measuring rate of 50 measurements per second of both the current and voltage leads to a high amount of information while retaining the precision and accuracy of the SMU at such low currents and voltages. By applying a small bias (1 mV) over the electrodes, seen in Figure 1c, one can *in situ* monitor whether a percolating network has been achieved between the electrodes, without causing any destruction to any poorly connected prepercolation conductive paths. At a bias voltage of 1 mV, the background noise measured before percolation is 8 ± 1 pA. By choosing the point to halt the deposition of NPs, one can choose between any initial resistance level of the network altering the behavior of the device. A high initial resistance deposition is demonstrated in Figure 1c, where switching behavior can be observed immediately by continuing to apply the 1 mV bias. In the Results and Discussion section, electrical measurements were performed on a sample that possesses a slightly lower resistance compared to that of Figure 1. Switching was then observed from a bias application of 5 mV and above. This switching behavior, however, is observed for all samples produced in the manner described above.

Postdeposition electrical analysis is performed inside the cluster source to omit any ambient air influences. This is achieved by performing IV-curve measurements and constant bias voltage applications, typically ranging from 1 to 100 mV.

### 4.3 Electrical Data Analysis

First, a brief conductance analysis is performed, as shown in Figure S3. It reveals not only different conductance levels but also changes of the conductance over time. Hereafter, analysis of the electrical data is performed on step sizes of the atomic conductance switches and temporal correlations using a spike-train analysis. Switches need to be identified using a threshold analysis as described here. First, a Savitzky-Golay filter is applied to reduce the potential identification of noise as switches. Regularly, however, there are single data point switches too fast to fully resolve with the above-mentioned measuring rate. Nonetheless, these are not filtered out and are considered as a switch event. Hereafter, the derivative is taken upon which timestamps of a switch event are revealed in the form of spikes. By identifying the extrema, above a certain threshold, with a peak finding algorithm, the timestamps of the switches can reliably be detected. These are then plotted in orange in raw-data plots at the conductance level of the timestamp. In Figure S4, the switch identification is depicted. To analyze step sizes, the difference between the previous and next conductance levels with respect to every event timestamp is calculated by taking the difference of the averages over the previous or next x data points, dependent on the distance to the next switch event. Typical power law fitting, meant to reveal spatial self-similarity in percolating structures,^19^ is shown in Figure S6. Interevent intervals (IEIs) can be calculated by subtracting the timestamps of consecutive switches to set up a probability density function (PDF). Autocorrelation functions (ACFs) can reveal, together with IEIs, whether avalanching effects are present in the networks.^19^ To both, physically representative models can be fitted, which can then be compared to neuronal and synaptic spiking behavior. The fitting is performed using a nonlinear least-squares method to minimize errors and fit the parameters within a 95% confidence interval.

### 4.4 (Scanning) Transmission Electron Microscopy

Using a probe- and image-corrected Thermo Fisher Scientific Themis Z (scanning) transmission electron microscope at 300 kV, the elemental constituents, morphology, and crystalline structures of the Mo NPs were characterized after deposition. Vacuum transfers were not an option, leading to contact with ambient conditions but with minimal effect (see SI: methods section). An example of the as-deposited Mo NPs can be seen in Figure 1d. In all (S)TEM images, the high-angle annular dark field (HAADF) (S)TEM detector has been utilized unless stated otherwise. The beam convergence angle was 25 mrad, and a probe current of 50 pA was used for (S)TEM imaging. Applying a simple area analysis of the (S)TEM image in Figure 1d, an area coverage of 67 ± 1% is found, approximating the percolation threshold for a well-based 2D continuum model well. Typically, percolation thresholds for such a model achieve percolation at an area coverage of approximately 67.6%.^38^ Finally, (S)TEM-energy dispersive X-ray spectroscopy (EDS) was used to analyze the elemental components of representative NPs. These results were obtained with a Bruker Dual-X EDS system using two large area detectors in total, capturing 1.76 steradians with a probe current of 450 pA for approximately 1.5 h. Data acquisition and analysis were performed using Velox software.

## Supporting Information Available

Complementary methods section on conductance analysis. Complementary methods section on effect of sample transfer to TEM. Complementary methods section on COMSOL Multiphysics simulations, including Figure S1. Complementary results section, supplying an overview of the measurement data including several more snapshots in Figure S2. Conductance analysis is shown in Figure S3. Switchfinding and its influence are shown in Figure S4 with accompanying results comparison between Figure 2 and Figure S5. Moreover, in Figure S6, the sizes of switches have been analyzed. A scanning electron microscopy image is shown in Figure S7 on NP shape representation. Herafter, in Figure S8 and Figure S9, COMSOL Multiphysics simulations are shown. Finally, in Figure S10, copper is compared with molybdenum, including an electrical measurement data set (PDF)
COMSOL simulations in all orientations are shown in a movie of Figure S9 (AVI)

## Supplementary Material

am4c12051_si_002.pdf

am4c12051_si_003.avi
