# Supplementary material for: Stable Millivolt Range Resistive Switching in Percolating Molybdenum Nanoparticle Networks
Source: ACS Appl Mater Interfaces. 2024 Nov 13;16(47):65157–64. doi: 10.1021/acsami.4c12051 (PMC11615851; doi:10.1021/acsami.4c12051)
Supplement: Supplementary file 1 — am4c12051_si_002.pdf [file am4c12051_si_002.pdf]

Supporting Information: Stable Millivolt Range Resistive Switching in Percolating Molybdenum Nanoparticle Networks

Adrianus Julien Theodoor van der Ree,[*,†,‡] Majid Ahmadi,[†] Gert H. Ten Brink,[†] Bart J. Kooi,[†] and George Palasantzas[*,†]

†*Zernike Institute for Advanced Materials, University of Groningen, 9747 AG Groningen, The Netherlands*

‡*CogniGron Center, University of Groningen, 9747 AG Groningen, The Netherlands*

E-mail: a.j.t.van.der.ree@rug.nl; g.palasantzas@rug.nl

Methods

Conductance Analysis

Conductance analyses have been performed on all 12-hour electrical measurements to analyze consistency and be able to find the major occupied conductance levels. The conductance over the complete measurements have been grouped in a square root of the amount of datapoints binning. Hereafter, to obtain the conductance levels observed in the histograms, a Gaussian mixture algorithm is implemented. A set of fitting parameters are introduced and all possible combinations are calculated, upon which the best representation of the data is chosen according to the Bayesian information criterion. This way does represent the data the best, but will not always seem proper from the individual Gaussian conductance levels. Nonetheless, the means of the conductance levels are approximated well. This can be observed in S3.

(Scanning) Transmission Electron Microscopy

After deposition, (scanning) transmission electron microscopy ((S)TEM) is performed. We stated that the samples are not transferred to the transmission electron microscope via a vacuum transfer. Rightfully so, it can be questioned whether the (S)TEM results, including the electron dispersive x-ray spectroscopy (EDS) are then still truthful. The oxide layer that can be observed in Fig. 3 is not formed in ambient conditions, but rather inside the vacuum. This cannot be proved directly without performing a transfer to the microscope in vacuum, but can be argued indirectly. The filamentary switching would not be present in vacuum, as the lack of an oxide layer will cause the NPs to coalesce, creating a well-conducting nanocrystalline film. Moreover, molybdenum is also known for its self-limiting diffusion layer. Once an oxide layer is grown on molybdenum (in physical vapor deposition), the oxide layer limits the diffusion of oxygen into the NP itself. This is indeed the case, as the switching is stable over long periods of time (at least days without significant changes).

Hence, whether the samples are exposed to ambient conditions or not, has no significant effect on the analyses performed in Fig. 3.

COMSOL Multiphysics® Finite Element Method electrostatics simulation

COMSOL Multiphysics® finite element method simulations can be very instrumental. Here, we utilize the simulation program to analyze electrostatic interactions between two stellate NPs. The simulation consists of two 20 nm in diameter NPs, at a constant (irrespective of orientation), center-to-center distance of 35 nm, with a potential difference of 5 mV over the two. The left NP supplies the bias whereas the right is the ground terminal. Both NPs are in a non-influential ambient vacuum space (as in the cluster source system), and are both set to have Mo material properties. A mesh is defined where the vacuum has an extremely coarse, physics-based, mesh, whereas the NPs require a much finer mesh. Here, a minimum element size was set to 0.3 nm, a fairly large maximum element growth rate of 4 (for reduction of the number of elements), and a curvature factor of 0.45 to keep sufficient resolution at the tips. With these parameters, a good balance between computation time (1-10 seconds per calculation) and trustworthy results is achieved. The respective meshes and an example potential distribution can be seen in Figure S1.

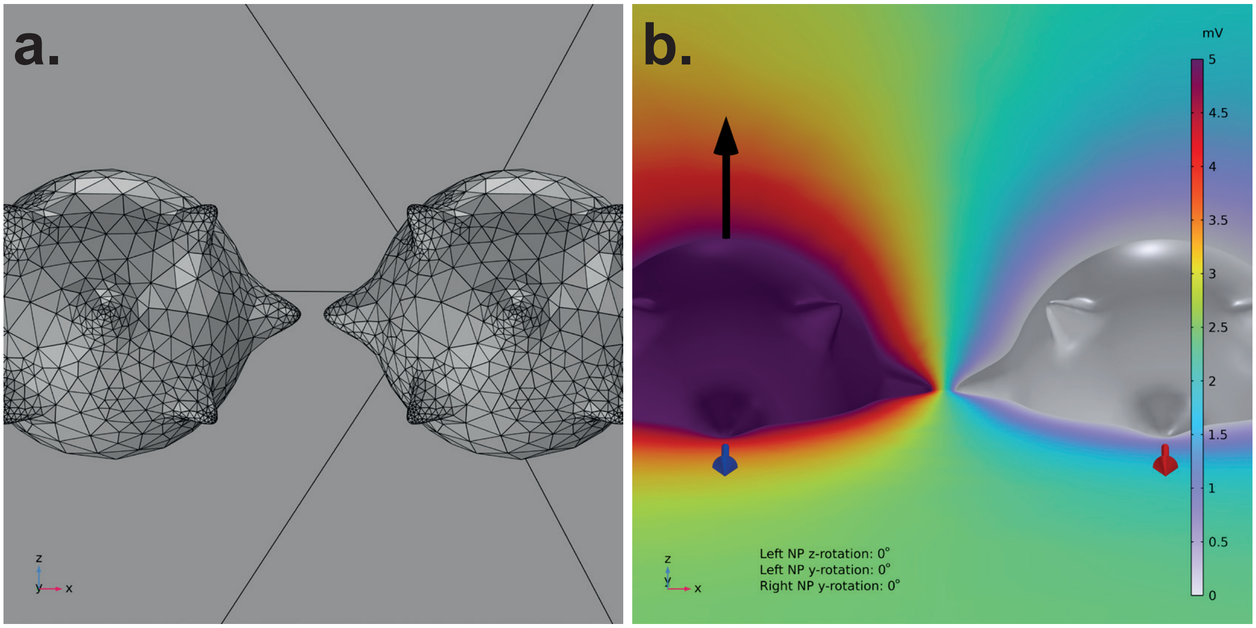

Figure S1: In **a.** the meshes of the NPs are shown, with the vacuum boundary mesh in the background, revealing the differences in element sizes. In **b.**, the potential distribution between the NPs is shown. The black, blue and red arrows represent the left NP z-, y- and right NP y-rotation axes respectively.

Results

Data over 12 hours

To provide more information on the switching dynamics over 12 hours, in Figure S2, an overview and 2 more sections of the dataset have been presented. As can be seen, switching dynamics are fairly consistent throughout the measurements. Noteworthy are the changes over several hours, in the overviews. These indicate different switching dynamics over time and to a certain extent, is true. However, it is important to treat the network as a whole over time, and not in sections, as these events can even happen when it might not be as obvious.

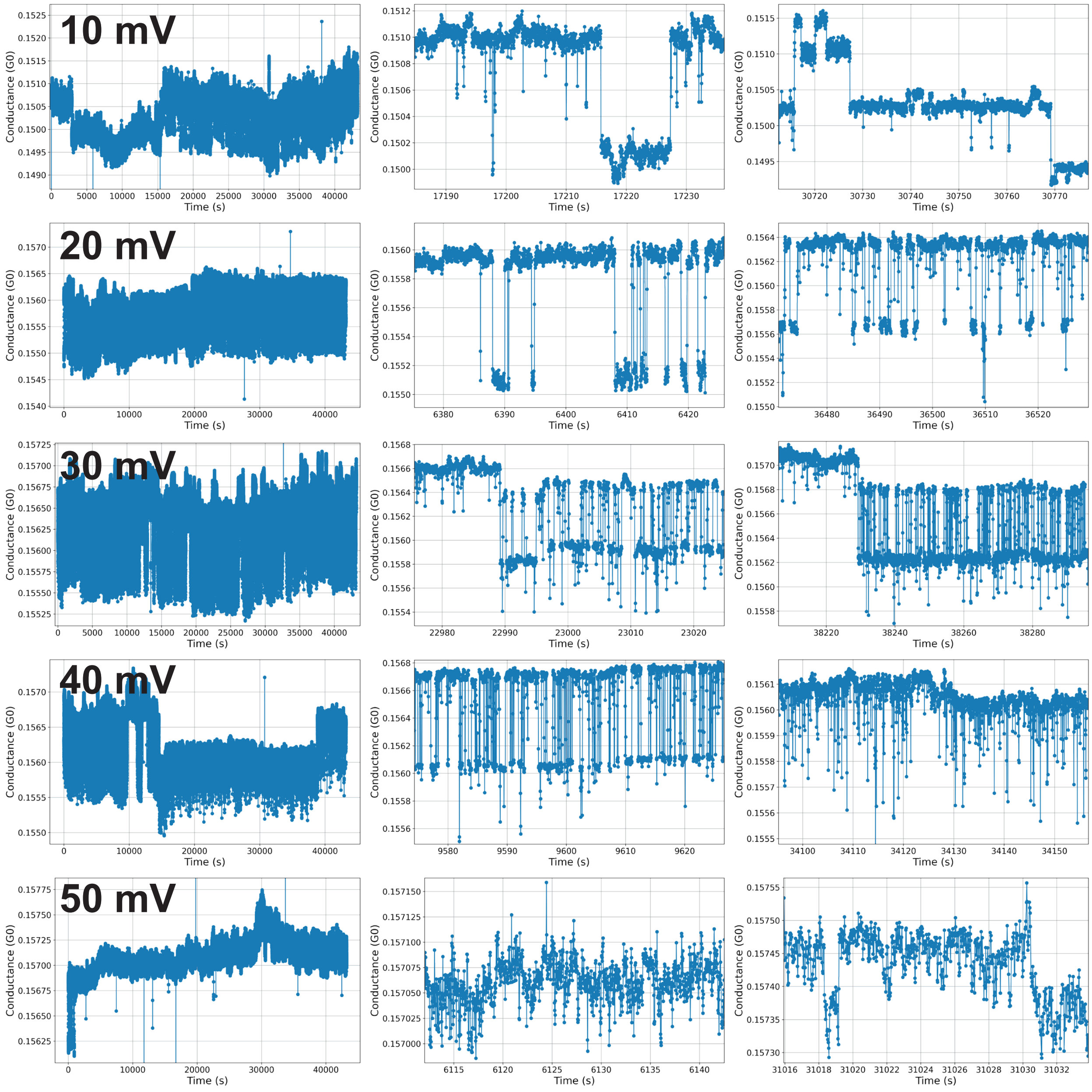

Figure S2: Overviews, paired with multiple zoomed in sections, are shown for each measurement in their respective rows. Switching dynamics are consistent throughout. The overview is the 12 hour measurement whereas the zoomed in subfigures represent ≈ 30-60 seconds.

In Figure S3, a 30 minute, 5 mV bias measurement can be seen along with the histograms of the 5 mV measurement and all 12-hour measurements. Observed are distinctive levels at which the conductance rests in between switching events. Over time, different levels will be occupied due to different filamentary switch locations being enabled. Over 12 hours,

the measurements are fairly stable in respect to the conductance levels. In FigureS3a., the 5 mV measurement is used illustratively, how the fitting is portrayed on the measurement data. The measurement includes switching, for example at a timestamp of 750 seconds, but minimal compared to the bias voltages beyond. Not all conductance levels that are occupied after a switching event will fall within one σ of the mean of a conductance level, which would only be observed clearly upon choosing a significantly, but less representative, smaller binsize. Nonetheless, the main conductance levels can be identified although there will be many more hidden within the Gaussian-fitted histograms.

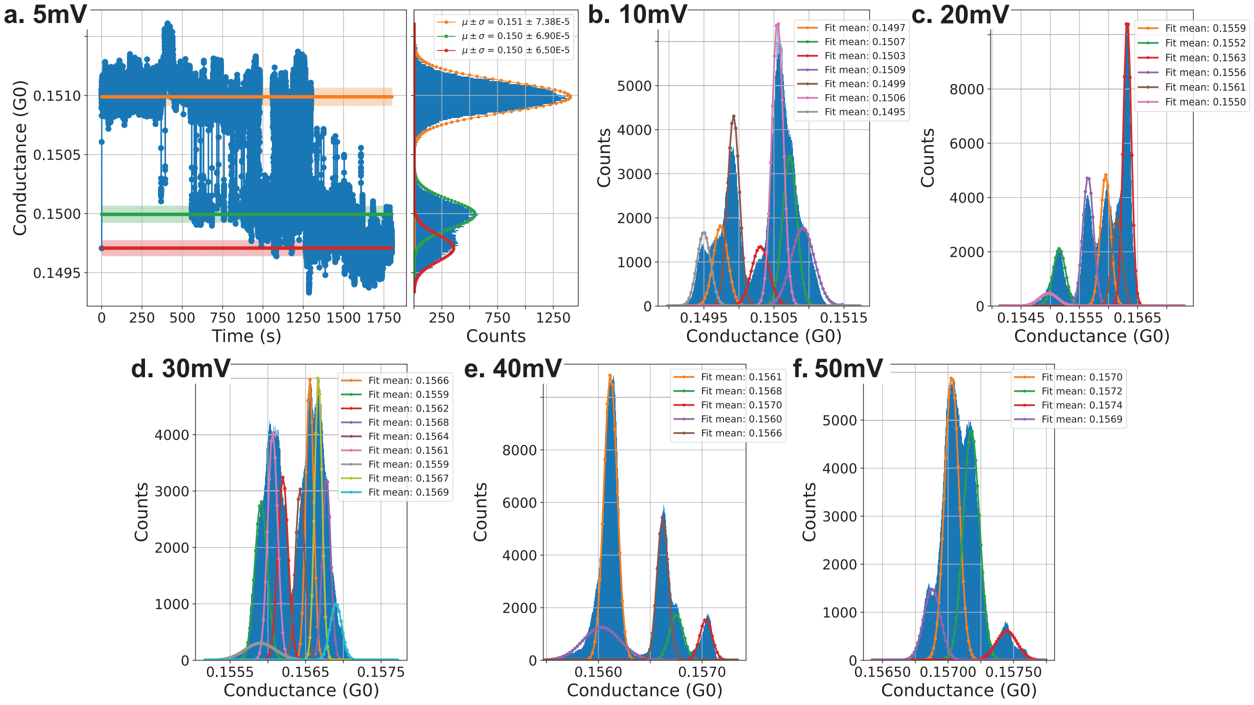

Figure S3: Histograms of all 12-hour measurements, paired with Gaussians for the respective conductance levels are shown in **b.-f.**. In **a.**, a 30-minute measurement at 5 mV bias voltage is shown, paired with its histogram of the different conductance levels. Note: the 50 mV measurement has less defined peaks due to measurement resolution and higher switching rate.

Switch finding and its influence

Analysis of the electrical data is performed on step sizes of the atomic conductance switches, and temporal correlations using a spike-train analysis. First, switches have been identified

using a threshold analysis as described here. First, a Savitzky-Golay filter is applied to reduce the potential identification of noise as switches. Regularly however, there are single data point switches too fast to fully resolve with the above mentioned measuring rate. Nonetheless, these are not filtered out and are considered as a switch event. Hereafter, the derivative is taken upon which timestamps of a switch event are revealed in the form of spikes. By identifying the extrema, above a certain threshold, with a peak finding algorithm, the timestamps of the switches can reliably be detected. These are then plotted in orange in raw-data plots at the conductance level of the timestamp. This process can be seen in Figure S4, where the original data is displayed, with its corresponding identified switch events. Moreover, the derivative of the filtered data can be seen in the bottom half of Figure S4. Note, the algorithm can pick out any switch, even those which are as short as a single data point.

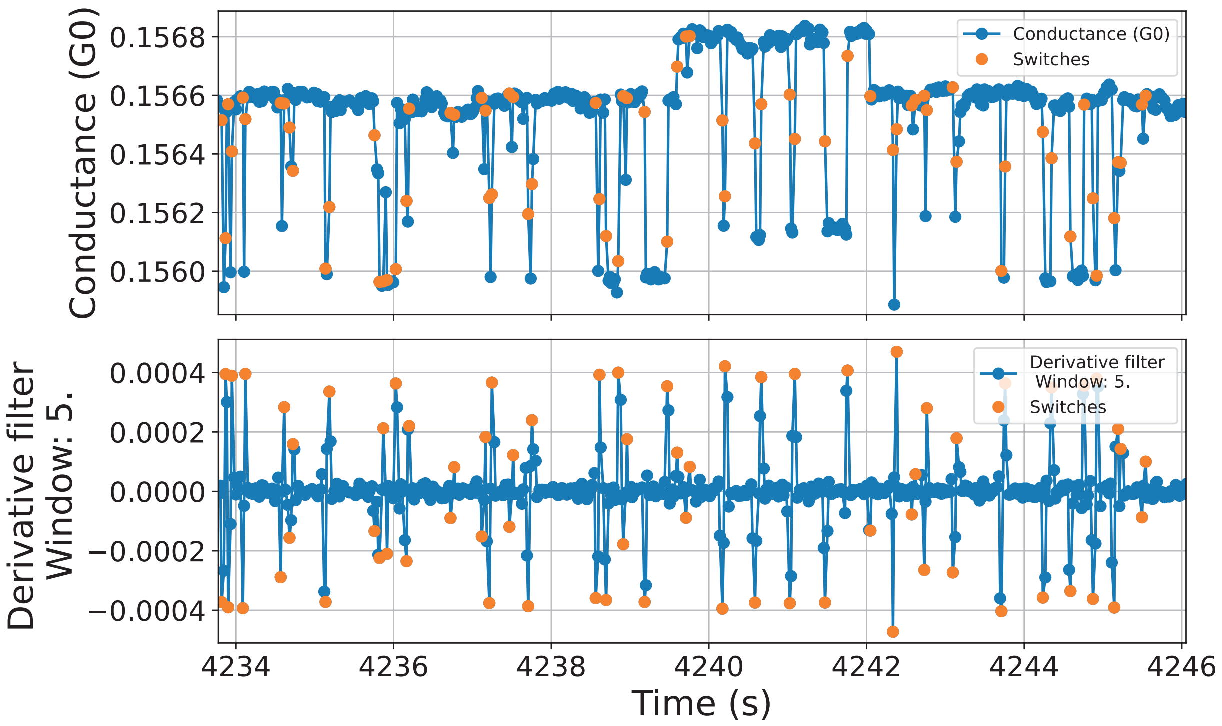

Figure S4: Here, the switch finding algorithm is displayed. In the top half, the conductance of a section of the 40 mV measurement is displayed, with its corresponding switch events. In the lower half, the derivative of the minimally filtered (5 point moving average) data is displayed, where the peak finding algorithm as found the peaks above a threshold of 5×10^{-5}.

Upon changing the threshold to a higher level, due to human error, or doubts arise regarding what an actual switch event is, the analyses definitely change slightly. This can be seen in Figure S5, where the threshold for the switch identification has been increased up until larger switches were still well resolved, and smaller were not.

The numbers of the fits decrease slightly in the cases for 10-40 mV, but still show a similar trend from bias to bias. The heavy tailed probability functions are still present, just like the autocorrelation functions still being clearly distinct from the shuffled IEI sequence ACFs. At 50 mV, it becomes very clear that switches are not being identified and has a clear effect on the analyses henceforth. Nonetheless, similar conclusions can be drawn from these results revealing that bursty, avalanche behavior is present.

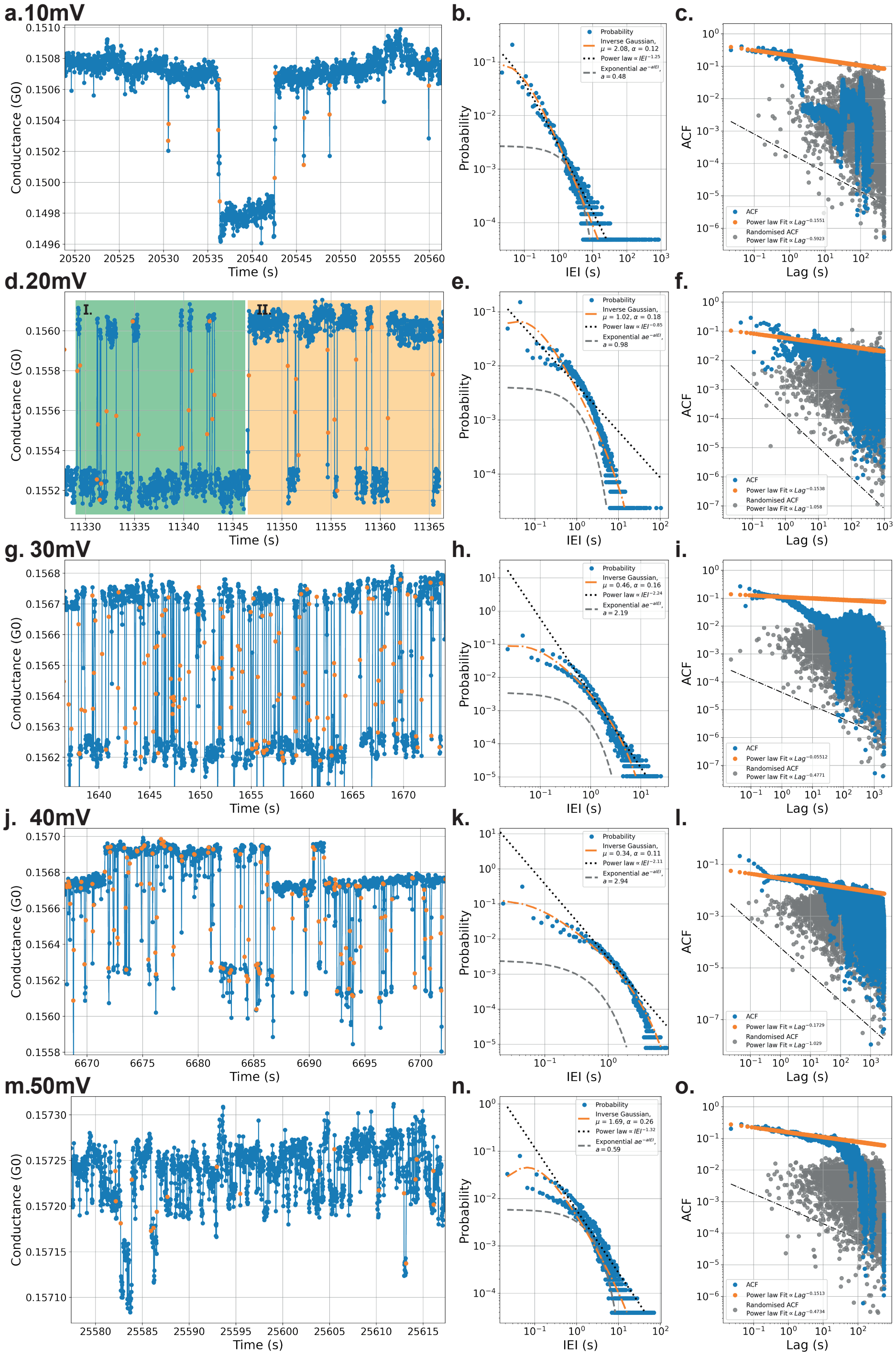

Figure S5: Here, the same analyses as in Figure 2 can be found. The difference lies in the increase in the threshold for switch finding, such that large, clear switches are clearly resolved and smaller are not. The identification thresholds are: 2, 2, 2, 1, 0.4×10^{-4} for 10-50 (**a.**, **d.**, **g.**, **j.** and **m.**) mV datasets, respectively. In the second (**b.**, **e.**, **h.**, **k.** and **n.**) column the respective IEIs are shown. Finally, in the third (**c.**, **f.**, **i.**, **l.** and **o.**) column, the respective ACFs can be found.

Switching step size analysis

To analyze step sizes, the difference between the previous and next conductance levels with respect to every event timestamp is calculated by taking the difference of the averages over the previous or next x data points, dependent on the distance to the next switch event. Typical power law fitting, meant to reveal spatial self-similarity in percolating structures, has been performed. The statistical analysis of the step sizes can be seen in Figure S6. The power law fits are not great fits due to the size of the network, and therefore having more individual switches compared to a large percolating NP network, as often observed with Paolo Milani and Simon Brown. This entails that there are more defined steps rather than a power-law distribution of steps. Moreover, the 50 mV measurement possessed switching activity too rapid to measure and hence, this is also seen in this plot where no reliable statistical data can be extracted.

Shape representation Mo NPs

To provide a little bit clearer information regarding the shape of Mo NPs, a low coverage Mo NP deposition is presented here. These NPs have been deposited onto a continuous carbon copper TEM grid, and have been imaged in a Helios G4 CX scanning electron microscope (SEM). The images were taken at the same time, where Figure S7a is imaged with a through lens detector, whereas Figure S7b is imaged with a transmission detector below the sample.

As can be observed, there are a few larger NPs (often produced during the onset of NP production), and many small ones of approximately 20 nm in diameter. More importantly, all of them have a stellate shape. When depositing higher coverage NP films, such details can become more lost in imaging, even though the shape is still there.

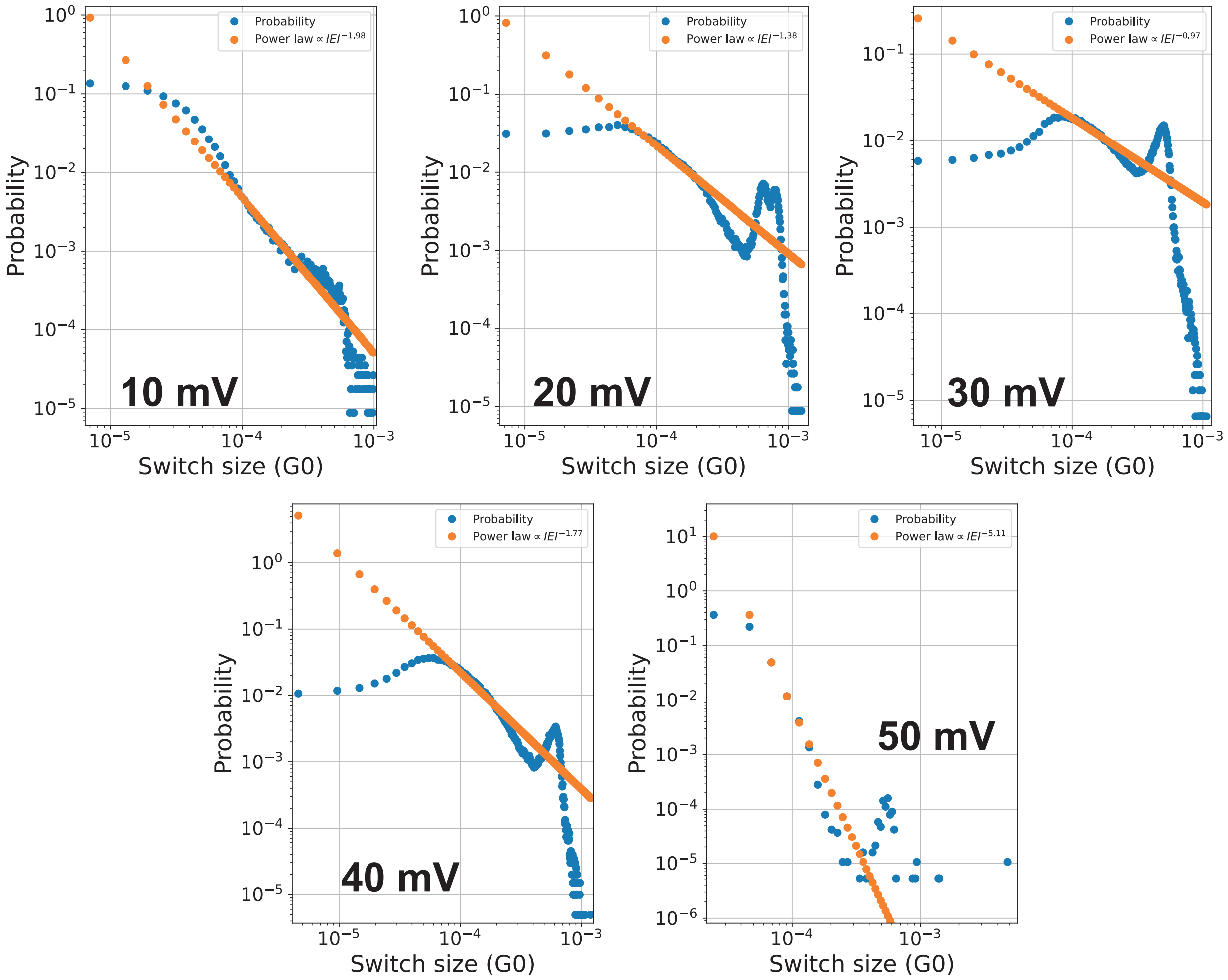

Figure S6: In the subfigures, each belonging to their individual 12 hour measurements, the probability distribution of the switch sizes are shown in blue. In orange, the power law fits have been plotted.

COMSOL Multiphysics® Finite Element Method electrostatics simulation

To investigate the influence of the morphology, a COMSOL Multiphysics® electrostatics simulation was set up for our stellate NPs and compared with purely spherical NPs, see the methods section for more details. In Figure S8, electrostatic simulations of differently oriented NPs are shown. Using two cross-sectional planes, streamlines, and NP surfaces, relatively high electric field strengths in between the biased NPs are highlighted in red concentrated areas. Compared to spherical NPs, one can observe large differences in both

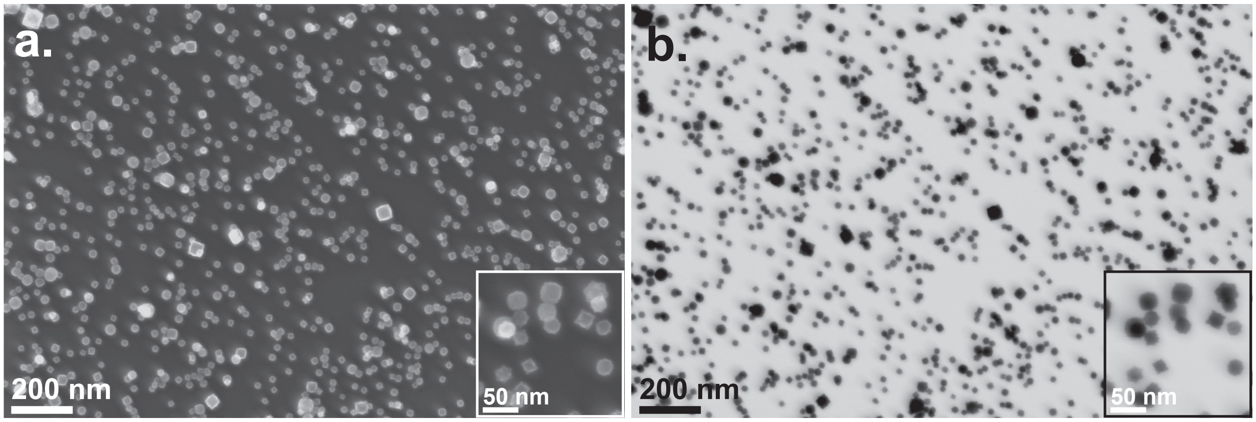

Figure S7: In **a.** and **b.**, an overview of a low coverage Mo NP film can be observed, imaged with a Helios G4 CX scanning electron microscope. **a.** is imaged with a through lens detector whereas **b.** is imaged with a transmission detector. In the insets is a zoomed in section of the images.

magnitude, but also distribution of the electric fields. The features of the NPs result in high electric field strengths at the tips in all orientations (see Movie S9), and they can facilitate electromigration well at lower voltage biases. Moreover, electromigration will therefore be dominant at the tips compared to the bulk of the NP, more so compared to spherical NPs. One could argue that spherical NPs are, therefore, also easier to coalesce and create more permanent connections due to the mass of the material which might electromigrate and hereafter need to be Joule heated to break apart again. By comparing these two morphologies, stellate NPs can have more consistent switching dynamics.

In Figure S9, a movie is supplied for different orientations of the two NPs. In every orientation, the protrusions possess the maximum field strengths, facilitating electromigration well.

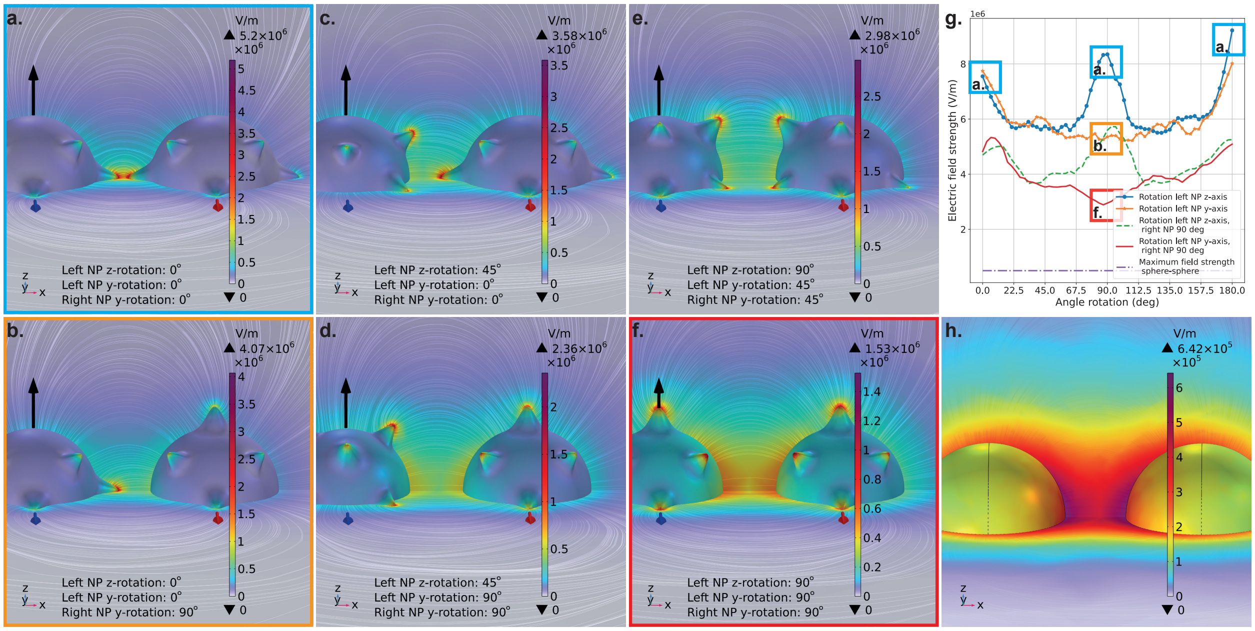

Figure S8: A COMSOL Multiphysics® electrostatics simulation, where between two NPs a 5mV bias is applied. To visualize the concentrations of the electric fields, the electric field strength is plotted in the xy- and xz-planes, paired with respective streamlines, and also on the surface of the NPs. In **a.-f.** different edge case orientations of two stellate Mo NPs are shown. The black, blue, and red arrows represent the left NP z-, y- and right NP y-rotation axes respectively, where the original state is indicated in **a.**. In **g.**, the maximum field strength versus different orientations of the NPs is plotted, including highlights for certain orientations, together with the maximum value for two spherical NPs, which can be seen in **h.**. Note: the color bars are different in magnitude to visualize electric field strengths clearly in every orientation. Comparisons in color values between the different plots cannot be made.

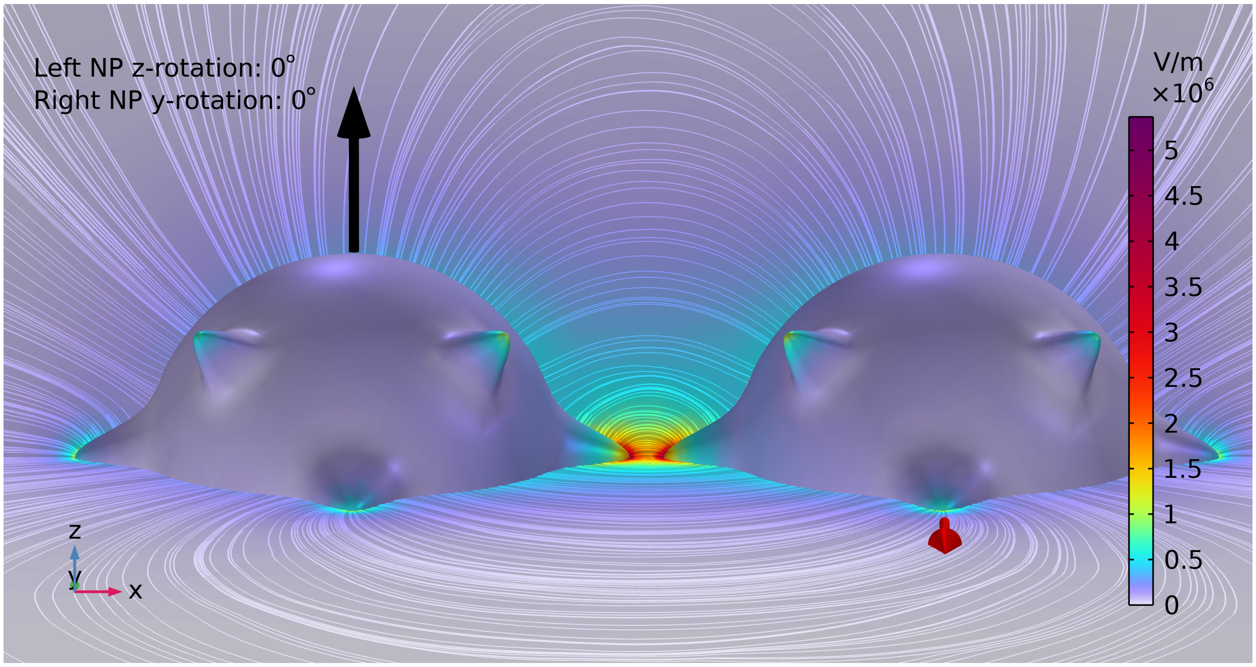

Figure S9: Effect of NP morphology on electric field concentrations in different orientations. Note: the color bar is synchronized, resulting in less apparent, yet still present, electric field concentrations in the spike features of the NPs.

Comparing molybdenum with copper NP networks

One aspect of the percolating Mo NP networks, is the switching dynamics. As can been seen in the electrical data, the switching is predominantly 2-level switching. This switching is very stable, and even over 12 hours, still extremely close to its original starting point. We believe this behavior can be attributed to the significant difference in our NP morphology compared to theirs. Characteristic for our as-deposited NPs is that they are stellate, whereas in earlier works the as-deposited NPs are spherical. This morphology aids in keeping NPs apart, and aids in reducing coalescence which, in turn, results in stable, consistent and different switching dynamics. To gain an insight into material differences, spherical, 12 nm diameter Cu NPs were the constituents of a different percolating NP network. Switching in percolating Cu NP networks required similar bias applications to induce switching but there were two key differences. See Figure S10. The Cu networks would regularly have permanent, large switch events, indicative of melting of the network, and, the average conductance would not stop to increase (even after significant efforts to omit this behavior), indicating substantial coalescence of the network. This is because both shape and material make coalescence and melting of the network significantly easier. This can be clearly seen from the differences in the NP appearances, where the Mo NPs are distinct and not coalesced, the Cu NPs are spherical, slightly fuzzy, but still more apart compared to a continuous film of NPs. The latter is due to an oxide film around the Cu NPs, keeping the NPs separated and the network switching.

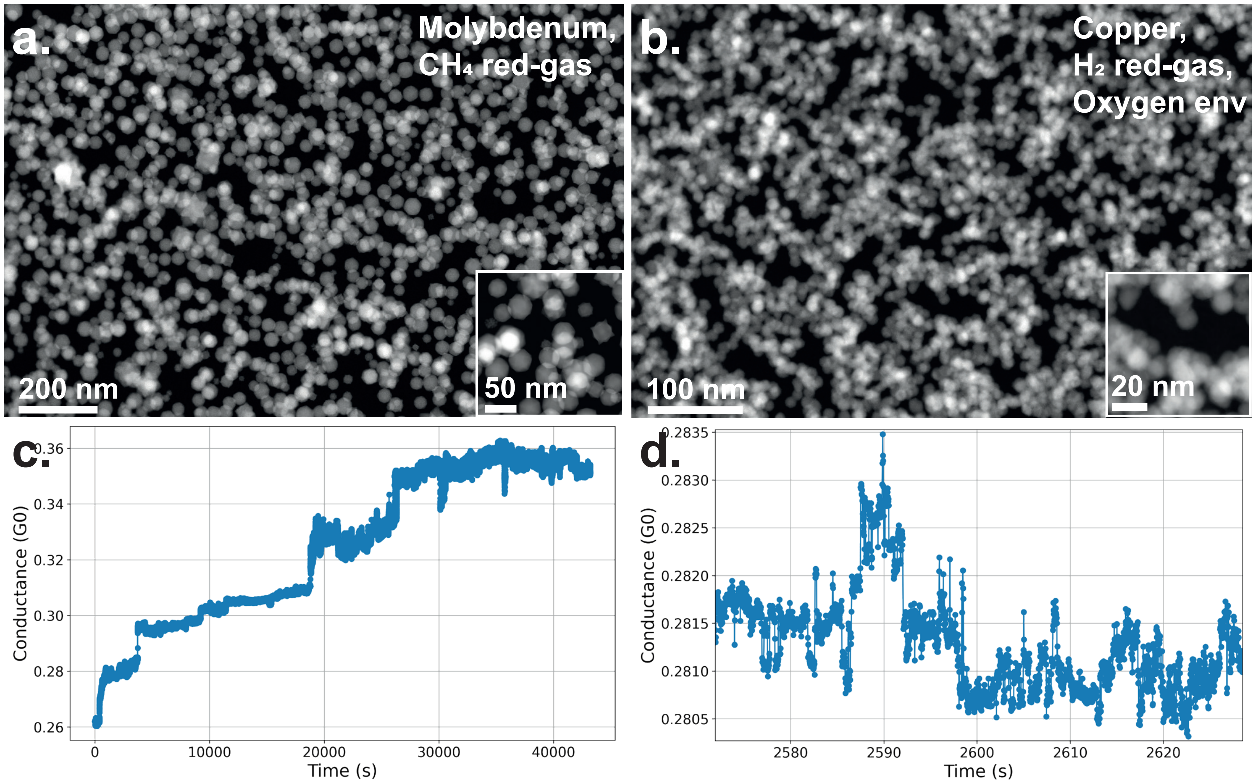

Figure S10: In **a.**, the percolating Mo NP network can be observed. The percolating Cu NP network can be observed in **b.**. The insets are zoomed in sections of the images. In **c.**, the conductance of the Cu NP network, biased at only 15 mV, over a 12 hour measurement. in **d.**, a section of the dataset is presented. Note, the switching is definitely not bi-level, nor the same as when the measurement started. Locally and overall, large changes are observed.
